# Prognostic models for mucinous and non-specific adeno cholangiocarcinoma: a population-based retrospective study

**DOI:** 10.3389/fendo.2024.1284283

**Published:** 2024-06-11

**Authors:** Muhammad Salman Azhar, Zi-jian Zhang, Zhong-tao Liu, Yun-peng Huang, Yong-xiang Wang, Hui Zhou, Li Xiong, Yu Wen, Heng Zou

**Affiliations:** Department of General Surgery, Second Xiangya Hospital, Central South University, Changsha, Hunan, China

**Keywords:** cholangiocarcinoma, pathologic subtype, risk score, prognosis, mucinous cholangiocarcinoma

## Abstract

**Background:**

Clinically, the diagnosis and treatment of cholangiocarcinoma are generally different according to the location of occurrence, and the studies rarely consider the differences between different pathological types. Cholangiocarcinomas in large- and middle-sized intrahepatic bile ducts are mostly mucinous, while in small sized bile duct are not; mucinous extrahepatic cholangiocarcinomas are also more common than mucinous intrahepatic cholangiocarcinoma. However, it is unclear whether these pathological type differences are related to the prognosis.

**Methods:**

Data of total 22509 patients was analyzed from Surveillance, Epidemiology, and End Results program database out of which 22299 patients were diagnosed with common adeno cholangiocarcinoma while 210 were diagnosed with mucinous cholangiocarcinoma. Based on the propensity score matching (PSM) analysis, between these two groups’ clinical, demographic, and therapeutic features were contrasted. The data were analyzed using Cox and LASSO regression analysis and Kaplan-Meier survival curves. Ultimately, overall survival (OS) and cancer specific survival (CSS) related prognostic models were established and validated in test and external datasets and nomograms were created to forecast these patients’ prognosis.

**Results:**

There was no difference in prognosis between mucinous cholangiocarcinoma and adeno cholangiocarcinoma. Therefore, we constructed prognostic model and nomogram that can be used for mucinous and adeno cholangiocarcinoma at the same time. By comparing the 9 independent key characteristics i.e. Age, tumor size, the number of primary tumors, AJCC stage, Grade, lymph node status, metastasis, surgery and chemotherapy, risk scores were calculated for each individual. By integrating these two pathological types in OS and CSS prognostic models, effective prognosis prediction results could be achieved in multiple datasets (OS: AUC 0.70–0.87; CSS: AUC 0.74–0.89).

**Conclusion:**

Age, tumor size, the number of primary tumors, AJCC stage, Grade, lymph node status, metastasis, surgery and chemotherapy are the independent prognostic factors in OS or CSS of the patients with mucinous and ordinary cholangiocarcinoma. Nomogram that can be used for mucinous and adeno cholangiocarcinoma at the same time is of significance in clinical practice and management of cholangiocarcinoma.

## 1 Introduction

Cholangiocarcinomas are a vast group of malignancies which are believed to have their origin in the biliary tract epithelium, either inside the liver or the biliary tract. These malignancies are usually hard to diagnose, their pathogenesis is not very well understood yet, their poor prognosis has been the main reason that their management has taken a nihilistic turn (1, 2). Cholangiocarcinoma is the collective term used for malignancies arising within the intrahepatic and extrahepatic biliary tract. A large proportion (90%) of cholangiocarcinomas are adenocarcinomas, with various already reported histological variants, including adenocarcinoma, papillary adenocarcinoma, intestinal-type adenocarcinoma, and mucinous adenocarcinoma (3). This huge difference of number lead cholangiocarcinomas to be classified broadly into two groups adenocarcinomas and ‘rare variants’ (4). Mucinous carcinoma presents with the presence of large extracellular mucus lakes which have floating cancer cells, accounting for more than half of neoplasm (5). Regardless of kind, it is unquestionably acknowledged that cholangiocarcinoma is a fatal cancer that typically manifests late, is famously difficult to identify, and is associated with a very high fatality rate. The occurrence of intrahepatic cholangiocarcinoma is increasing around the globe (6–10). The reason for this increase is not clear, although it could be related to an interplay between predisposing genetic factors and environmental triggers. MRI and CT with endoscopic ultrasound and PET has made it easier to get the important diagnostic information in certain group of patients. Surgical resection of the tumor is the only chance for cure, with results depending on personal surgical skills of the surgeon and patient selection. Studies have reported that liver transplantation could be a choice for long-term survival in selected patients when combined with neoadjuvant chemoradiotherapy. Chemotherapy and radiotherapy have been ineffective for patients which had unresectable tumors (11). As the global incidence has remarkably increased, mortality is high, treatment options are very few, prognosis is poor and overall survival is not promising (12, 13), clinical practice is truly in need of a method to calculate the survival probability of these patients based on different prognostic factors. Analyzing mucinous and non-specific adenocarcinoma subtypes within cholangiocarcinoma is crucial for comprehensively understanding the disease spectrum, guiding clinical decision-making, and improving patient outcomes. This approach underscores the importance of a multidisciplinary approach integrating histopathological, molecular, and clinical data to refine disease classification and treatment strategies. But unfortunately, currently available studies focused on limited types and didn’t cover the rare variants. So our study focused on analyzing and establishing a prognostic model which will give insights about both adenocarcinomas and mucinous cholangiocarcinomas.

Developed in the United States, the Surveillance, Epidemiology and End Results (SEER) database contains population-based clinical survival information from registries that cover 34.6% of the country’s population. We therefore concentrated on creating a predictive nomogram for adenocarcinomas as well as an uncommon variation, mucinous cholangiocarcinoma, in this work based on statistics from the SEER database.

## 2 Materials and methods

### 2.1 Data source and case selection

The National Cancer Institute’s SEER cancer database (http://www.seer.cancer.gov), which was released in November 2021, was used to extract information about patients with cholangiocarcinoma (version 8.4.0.1). In the United States, 17 population-based cancer registries provide information on cancer that is gathered by the SEER program. Because the data in the SEER database was de-identified and tagged for public availability, the data from SEER database was relieved from the necessity to get ethical approval from the Second Xiangya Hospital of Central South University Evaluation Board.

**Figure 1:**
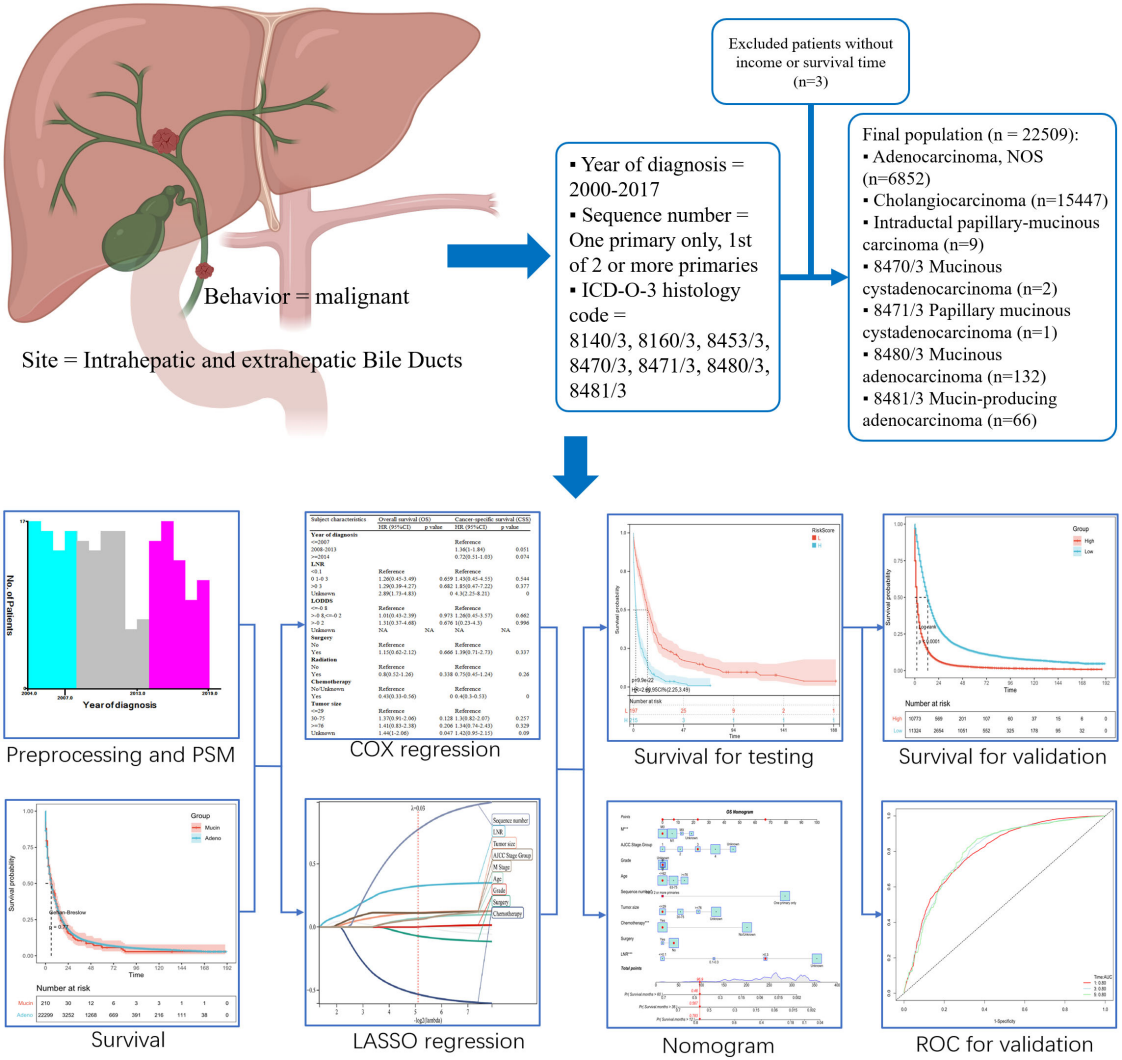
Study flow chart.

Using the use of the site-specific International Classification of Oncological Diseases 3 (ICD-O-3) code, C221, C240, C248, C249, cholangiocarcinoma in the SEER program was recognized from 1998 to 2016. The diagnosis of adenocarcinoma or cholangiocarcinoma (hereinafter referred to as adeno cholangiocarcinoma) was made using the ICD-O-3 codes 8140/3 and 8160/3, while the diagnosis of mucinous cholangiocarcinoma was made using the ICD-O-3 codes 8453/3 (intraductal papillary-mucinous carcinoma), 8470/3 (mucinous cystadenocarcinoma), 8471/3(papillary mucinous cystadenocarcinoma), 8480/3(mucinous adenocarcinoma), 8481/3 (mucin-producing adenocarcinoma). Using the sequence number for a single primary tumor or the first of two or more primaries, the number of primary tumors was determined. The following were the exclusion criteria: (1) the tumor was not primary; and (2) the case lacked full follow-up information or household income. The final group of patients included 22299 with adeno cholangiocarcinoma and 210 with mucinous cholangiocarcinoma. The standards of the SEER database coding and staging manual were followed for creating the codes for case collection (**Figure 1**).

### 2.2 Clinical information acquisition and processing

Demographic data (such as patient ID and age), tumor characteristics (such as tumor size, the number of primary tumors, AJCC stage group, tumor grade, metastasis status, and lymph node status), treatment data (such as surgery, radiotherapy, and chemotherapy status), and follow-up for survival were all extracted from the SEER database (survival months, cause-specific death, and vital status).

Age was used as a demographic indicator (≤62 years, 63–75 years, and ≥76 years). Tumor size (≤29mm, 30mm–75mm, ≥76mm, and unknown), the number of primary tumors (one primary only versus the first of two or more primaries), the AJCC stage (I/II, III/IV, or unknown), the grade (I/II, III/IV, or unknown), the status of the lymph nodes (negative, positive, or unknown), and metastasis were all noted as characteristics of the tumor (M0, M1, Mx or unknown). Surgery (yes or no) and chemotherapy (yes or no/unknown) were the two types of treatment.

**Figure 2:**
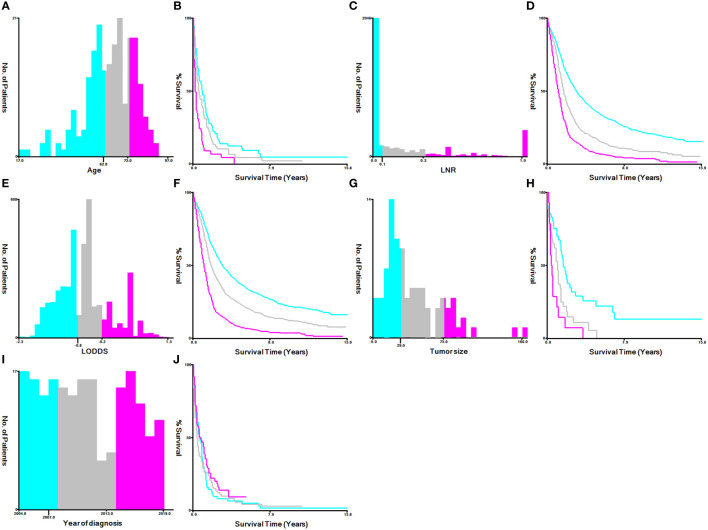
Identification of optimal cutoff values for the year of diagnosis, age, LNR, LODDS and tumor size via X-tile software analysis. **(A, B)** Optimal minimum and maximum cutoff values for age: 62 and 75 years and survival curve. **(C, D)** Optimal minimum and maximum cutoff values: 0.1~0.3 for LNR and survival curves. **(E, F)** Optimal minimum and maximum cutoff values: -0.8~-0.2 for LODDS and survival curves. **(G, H)** Optimal minimum and maximum cutoff values: 29 and 75 mm for tumor size and survival curves. **(I, J)** Optimal minimum and maximum cutoff values: 2007 and 2013 for the years of diagnosis and survival curves.

The best cutoff value for transforming continuous variables (such as age at diagnosis, year of diagnosis, tumor size, LNR, and LODDS) into categorical variables was determined using the X-tile program version 3.6.1. The varying age at diagnosis was next divided into three groups: 62 years and younger, 63 to 75 years, and 76 years and older. LNR was classified as ≤0.1, 0.1–0.3, >0.3, and unknown, LODDS as ≤-0.8, -0.2–0.8, >-0.2, and tumor size as ≤29mm, 30–75mm, ≥76mm, and unknown groups. The variable year of diagnosis was grouped as <2007, 2008–2013, and >2013 (**Figure 2**).

### 2.3 Survival analysis before and after the propensity score matching

Propensity score matching (PSM) was used to modify the baseline characteristics of patients with mucinous and adeno cholangiocarcinoma because the clinical characteristics between cases with mucinous and adeno cholangiocarcinoma in the SEER database were heterogeneous via the analysis of Chi-square or Fisher’s exact tests. The following PSM settings were carried out using the R package “MatchIt” version 4.1.0: 1-to-1 pairing, nearest neighbor approaches, and a caliper of 0.05. All of the aforementioned factors were included in the propensity score model.

The time from diagnosis to death from any cause is known as overall survival (OS). The time between a cholangiocarcinoma diagnosis and death was called “cancer-specific survival” (CSS). These two indices served as the study’s result endpoints. The Kaplan-Meier analysis was used to create the survival plot, and the log-rank test was used to compare patients with mucinous and adeno cholangiocarcinoma before and after PSM.

### 2.4 COX and LASSO regression for prognostic models and validation

The possible prognostic factors on the OS and CSS of patients with cholangiocarcinoma were identified using univariate and multivariate Cox regression models.

Least absolute shrinkage and selection operator (LASSO)-COX analyses were conducted to identify the ideal weighting coefficients for these features and construct a model to test whether various pathological types and clinical traits can collectively predict the prognosis of patients with mucinous and adeno cholangiocarcinoma after PSM. Using the R software’s ‘glmnet’ package, ten-fold cross-validation was used to create LASSO-COX regression models for OS and CSS. Moreover, in OS and CSS, the lambda parameter’s ideal values were 0.29 and 0.022, respectively.

Using the ‘pROC’ package in R, ROC analysis of the follow-up outcomes and risk scores over ten years was conducted based on the aforementioned model, and the area under the curve (AUC) and confidence interval were analyzed (CI). Patients were divided into high- and low-risk groups based on the ideal cutoff or median of risk scores, and the prognostic differences between the two groups were further evaluated using the “survival” software. The log-rank test was used to determine the significance of the prognostic difference between the two groups.

As for validation, we first validated the model by using PSM excluded samples in the SEER database (n=22097). Furthermore, the study validated the OS and CSS prognostic models through an external validation cohort, which included 94 patients with cholangiocarcinoma from the Second Xiangya Hospital of Central South University. This study was conducted in accordance with the Declaration of Helsinki and approved by the Ethics Committee of Xiangya Second Hospital, Central South University (Changsha, China) with the informed consent of all patients. All patients were divided into high-risk and low-risk groups based on the optimal cutoff risk score calculated by the prognostic models. The Kaplan-Meier analysis and the log-rank test were used to examine differences in survival between the two groups. The ROC was used to determine the accuracy and discriminability of the prediction model.

### 2.5 Nomogram

Finally, the R ‘rms’ package was used to incorporate the survival data from the LASSO-COX analysis to create nomograms that predicted the OS and CSS of patients at 1, 3, and 5 years. A nomogram quickly determines the risk of sickness or the likelihood that an individual will survive by integrating numerous predictors and showing multiple lines to scale. The C-index is used to gauge the power of the nomogram.

## 3 Results

### 3.1 Comparison of the baseline clinical characteristics between mucinous and adeno cholangiocarcinoma

A total of 22509 patients including 22299 with adeno cholangiocarcinoma and 210 with mucinous cholangiocarcinoma in the SEER database, were enrolled in the study (**Figure 1**). The differences in most of the baseline clinical characteristics between mucinous and adeno cholangiocarcinoma e.g. age, year of diagnosis, LNR, LODDS, tumor size, sequence number, summary stage, AJCC stage group were statistically significant (p <0.05) (**Table 1**). Surgery status, chemotherapy, total number of tumors, race, median house hold income and marital of the patients with mucinous cholangiocarcinoma were similar to those with adeno cholangiocarcinoma (P>0.05).

**Table 1:** Demographic and clinical characteristics comparing adeno and mucinous cholangiocarcinoma (pre-PSM and post-PSM).

Subject	Before propensity score matching				After propensity score matching			
Characteristic	Mucin	Adeno	p value	SMD	Mucin	Adeno	p value	SMD
	N (%)	N (%)			N (%)	N (%)		
**All**	210	22299			206	206		
Year of diagnosis								
<=2007	63 (30.0)	3668 (16.4)	<0.001	0.366	59 (28.6)	52 (25.2)	0.566	0.105
>2013	73 (34.8)	10983 (49.3)			73 (35.4)	70 (34.0)		
2008-2013	74 (35.2)	7648 (34.3)			74 (35.9)	84 (40.8)		
LNR								
<=0.1	25 (11.9)	2322 (10.4)	<0.001	0.299	25 (12.1)	25 (12.1)	0.964	0.052
>0.3	23 (11.0)	920 (4.1)			20 (9.7)	21 (10.2)		
0.1-0.3	9 (4.3)	542 (2.4)			9 (4.4)	7 (3.4)		
Unknown	153(72.9)	18515 (83.0)			152 (73.8)	153 (74.3)		
LODDS								
-0.2~-0.8	20 (9.5)	1340 (6.0)	<0.001	0.295	20 (9.7)	21 (10.2)	0.985	0.039
<=-0.8	17 (8.1)	1660 (7.4)			17 (8.3)	17 (8.3)		
>-0.2	20 (9.5)	784 (3.5)			17 (8.3)	15 (7.3)		
Unknown	153(72.9)	18515 (83.0)			152 (73.8)	153 (74.3)		
Surgery								
No	193(91.9)	20975 (94.1)	0.243	0.085	190 (92.2)	189 (91.7)	1	0.018
Yes	17 (8.1)	1324 (5.9)			16 (7.8)	17 (8.3)		
Radiation								
No/Unknown	180(85.7)	19137 (85.8)	1	0.003	177 (85.9)	175 (85.0)	0.889	0.028
Yes	30 (14.3)	3162 (14.2)			29 (14.1)	31 (15.0)		
Chemotherapy								
No/Unknown	109(51.9)	12359 (55.4)	0.341	0.071	107 (51.9)	100 (48.5)	0.554	0.068
Yes	101(48.1)	9940 (44.6)			99 (48.1)	106 (51.5)		
Tumor size								
<=29	41 (19.5)	3731 (16.7)	0.015	0.242	40 (19.4)	50 (24.3)	0.231	0.205
>=76	14 (6.7)	2749 (12.3)			14 (6.8)	17 (8.3)		
30-75	35 (16.7)	4683 (21.0)			33 (16.0)	41 (19.9)		
Unknown	120(57.1)	11136 (49.9)			119 (57.8)	98 (47.6)		
Sequence number								
1st of 2 or more primaries	8 (3.8)	747 (3.3)	0.861	0.025	7 (3.4)	10 (4.9)	0.62	0.073
One primary only	202(96.2)	21552 (96.7)			199 (96.6)	196 (95.1)		
Total number of tumors								
1	203(96.7)	21663 (97.1)	0.913	0.058	200 (97.1)	197 (95.6)	NaN	0.082
2	6 (2.9)	585 (2.6)			5 (2.4)	7 (3.4)		
3	1 (0.5)	43 (0.2)			1 (0.5)	2 (1.0)		
4	0 (0.0)	4 (0.0)			0 (0.0)	0 (0.0)		
5	0 (0.0)	4 (0.0)			0 (0.0)	0 (0.0)		
Race								
Black	14 (6.7)	1841 (8.3)	0.138	0.132	14 (6.8)	22 (10.7)	0.236	0.168
Other	40 (19.0)	3216 (14.4)			37 (18.0)	43 (20.9)		
White	156(74.3)	17242 (77.3)			155 (75.2)	141 (68.4)		
Marital status								
DSW	48 (22.9)	5917 (26.5)	0.493	0.109	48 (23.3)	37 (18.0)	0.526	0.147
Married	126(60.0)	12226 (54.8)			122 (59.2)	126 (61.2)		
Single	28 (13.3)	3313 (14.9)			28 (13.6)	32 (15.5)		
Unknown	8 (3.8)	843 (3.8)			8 (3.9)	11 (5.3)		
Median household income								
<50,000	24 (11.4)	2685 (12.0)	0.788	0.048	23 (11.2)	28 (13.6)	0.696	0.084
>70,000	83 (39.5)	9208 (41.3)			81 (39.3)	75 (36.4)		
50,000-70,000	103(49.0)	10406 (46.7)			102 (49.5)	103 (50.0)		
Age								
<=62	87 (41.4)	7131 (32.0)	0.013	0.2	84 (40.8)	84 (40.8)	0.288	0.156
>=76	53 (25.2)	6875 (30.8)			53 (25.7)	41 (19.9)		
63-75	70 (33.3)	8293 (37.2)			69 (33.5)	81 (39.3)		
Grade								
I	20 (9.5)	739 (3.3)	<0.001	0.329	18 (8.7)	18 (8.7)	0.233	0.234
II	40 (19.0)	3088 (13.8)			39 (18.9)	31 (15.0)		
III	28 (13.3)	2841 (12.7)			28 (13.6)	44 (21.4)		
IV	2 (1.0)	95 (0.4)			2 (1.0)	4 (1.9)		
Unknown	120(57.1)	15536 (69.7)			119 (57.8)	109 (52.9)		
Summary stage								
Distant	27 (12.9)	3319 (14.9)	0.02	0.236	27 (13.1)	21 (10.2)	0.801	0.099
Localized	9 (4.3)	1754 (7.9)			9 (4.4)	11 (5.3)		
Regional	14 (6.7)	2366 (10.6)			14 (6.8)	14 (6.8)		
Unknown	160(76.2)	14860 (66.6)			156 (75.7)	160 (77.7)		
AJCC Stage Group								
1	22 (10.5)	3139 (14.1)	0.041	0.219	22 (10.7)	29 (14.1)	0.46	0.188
2	24 (11.4)	2978 (13.4)			24 (11.7)	25 (12.1)		
3	26 (12.4)	2618 (11.7)			25 (12.1)	28 (13.6)		
4	97 (46.2)	8129 (36.5)			94 (45.6)	96 (46.6)		
Unknown	41 (19.5)	5435 (24.4)			41 (19.9)	28 (13.6)		
T								
T0	2 (1.0)	188 (0.8)	0.052	0.246	2 (1.0)	3 (1.5)	0.24	0.281
T1	29 (13.8)	4478 (20.1)			29 (14.1)	35 (17.0)		
T2	21 (10.0)	2753 (12.3)			21 (10.2)	20 (9.7)		
T3	52 (24.8)	4172 (18.7)			50 (24.3)	50 (24.3)		
T4	27 (12.9)	2394 (10.7)			25 (12.1)	40 (19.4)		
TX	57 (27.1)	6597 (29.6)			57 (27.7)	45 (21.8)		
Unknown	22 (10.5)	1717 (7.7)			22 (10.7)	13 (6.3)		
N								
N0	82 (39.0)	10960 (49.2)	0.023	0.226	81 (39.3)	88 (42.7)	0.606	0.163
N1	52 (24.8)	4641 (20.8)			49 (23.8)	48 (23.3)		
N2	8 (3.8)	461 (2.1)			8 (3.9)	9 (4.4)		
NX	46 (21.9)	4529 (20.3)			46 (22.3)	48 (23.3)		
Unknown	22 (10.5)	1708 (7.7)			22 (10.7)	13 (6.3)		
M								
M0	81 (38.6)	10886 (48.8)	<0.001	0.299	80 (38.8)	88 (42.7)	0.429	0.165
M1	97 (46.2)	7761 (34.8)			94 (45.6)	96 (46.6)		
MX	10 (4.8)	1935 (8.7)			10 (4.9)	9 (4.4)		
Unknown	22 (10.5)	1717 (7.7)			22 (10.7)	13 (6.3)		

### 3.2 Propensity score matching and survival analysis

The PSM method was used to balance the baseline clinical characteristics between patients with mucinous and adeno cholangiocarcinoma (all standard deviations ≤ 0.05; **Table 1**). Patients were first classified into AD and MUC patient groups, and then logistic regression was used to calculate the probability of MUC for each patient with MUC as the dependent variable (Y) and all other known clinical characteristics as the independent variable (X). We then matched the experimental and control groups according to probability. In the actual software operation process, you need to provide a caliper value (caliper), caliper value is the experimental group and the control group in the matching, the probability of the allowable error, the caliper value of this study is 0.05.MUC and AD were paired according to this caliper value range in a 1: 1 ratio. In the last resort, 412 patients (mucinous group versus adeno group = 1: 1) were included in the following analysis after PSM.

**Figure 3:**
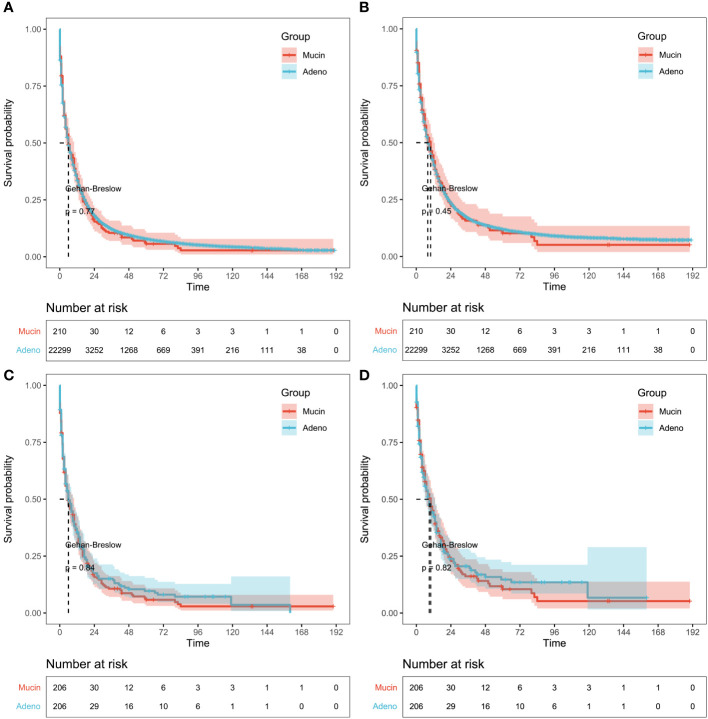
Survival results in pre-propensity score matching (PSM) and post-PSM. **(A)** Kaplan-Meier analysis of overall survival (OS) or **(B)** cancer-specific survival (CSS) based on patients with mucinous and adeno cholangiocarcinoma before PSM. Gehan-Breslow tests used to calculate P-values. **(C)** Survival analysis of OS or **(D)** CSS based on patients with mucinous and adeno cholangiocarcinoma after PSM. Log-rank tests used to calculate P-values.

Patients with mucinous and adeno cholangiocarcinoma pre- and post-PSM were appraised by Kaplan-Meier analysis. A total of 22509 patients including 22299 with adeno cholangiocarcinoma and 210 with mucinous cholangiocarcinoma were enrolled in the analysis before PSM. The median OS was 2.0 months in the mucinous and adeno group, while the median CSS was 2 months in the adeno group and 3.0 months in the mucinous group. So, no significant difference in outcome was observed in both groups. A similar result could be obtained for patients with mucinous and adeno cholangiocarcinoma after PSM. The median OS was 2.0 months in the mucinous and adeno group, while the median CSS was 2.0 and 3.0 months in the adeno group and mucinous group, respectively (**Figures 3A–D**) (1-year OS rates: 34.86% versus 35.51%, 3-year OS rates: 10.70% versus 15.06%, and 5-year OS rates: 5.79% versus 9.66%; 1-year CSS rates: 43.04% versus 42.46%, 3-year CSS rates: 16.14% versus 20.56%, and 5-year CSS rates: 10.46% versus 14.71%, after PSM, p > 0.05).

### 3.3 Univariate and multivariate analysis

Univariate and multivariate Cox regression analyses were performed to sift out the potential clinical characteristics of patients with mucinous cholangiocarcinoma which may influence the prognosis. In univariate regression analysis, age, year of diagnosis, LNR, LODDS, surgery, chemotherapy, tumor size, sequence number, tumor grade, summary stage, AJCC stage group and marital status were prognostic risk factors for both OS and CSS in patients with mucinous cholangiocarcinoma. (p <0.05, **Table 2**).

**Table 2:** Univariable Cox Regression for analyzing the associated factors for mucinous cholangiocarcinoma.

Subject characteristics	Overall survival (OS)		Cancer-specific survival (CSS)	
	HR (95%CI)	p value	HR (95%CI)	p value
Group				
Mucin	Reference		Reference	
Adeno	0.95 (0.77-1.16)	0.607	0.97 (0.78-1.22)	0.823
Year of diagnosis				
<=2007	Reference		Reference	
2008-2013	0.97 (0.76-1.25)	0.817	1.01 (0.77-1.33)	0.95
>=2014	0.78 (0.6-1.02)	0.075	0.73 (0.54-0.99)	0.044
LNR				
<=0.1	Reference		Reference	
0.1-0.3	1.2 (0.59-2.45)	0.608	1.75 (0.8-3.84)	0.162
>0.3	1.75 (1.08-2.84)	0.022	2.35 (1.32-4.16)	0.004
Unknown	3.43 (2.37-4.94)	0	4.66 (2.95-7.36)	0
LODDS				
<=-0.8	Reference		Reference	
>-0.8,<=-0.2	0.96 (0.55-1.68)	0.894	1.47 (0.75-2.87)	0.257
>-0.2	1.77 (1.03-3.03)	0.039	2.43 (1.25-4.71)	0.009
Unknown	3.16 (2.09-4.77)	0	4.62 (2.71-7.87)	0
Surgery				
No	Reference		Reference	
Yes	0.42 (0.28-0.64)	0	0.47 (0.3-0.72)	0.001
Radiation				
No/Unknown	Reference		Reference	
Yes	0.58 (0.43-0.79)	0	0.61 (0.44-0.85)	0.003
Chemotherapy				
No/Unknown	Reference		Reference	
Yes	0.61 (0.5-0.75)	0	0.62 (0.49-0.78)	0
Tumor size				
<=29	Reference		Reference	
30-75	1.79 (1.27-2.54)	0.001	1.93 (1.3-2.87)	0.001
>=76	2.1 (1.33-3.3)	0.001	2.43 (1.47-4.02)	0.001
Unknown	2.6 (1.96-3.45)	0	2.96 (2.14-4.09)	0
Sequence number				
1st of 2 or more primaries	Reference		Reference	
One primary only	2.34 (1.31-4.18)	0.004	3.28 (1.54-6.98)	0.002
Total number of tumors				
1	Reference		Reference	
2	0.27 (0.07-1.08)	0.064	0 (0-Inf)	0.991
3	0.79 (0.3-2.03)	0.618	0 (0-Inf)	0.992
Race				
Black	Reference		Reference	
Other	0.67 (0.44-1.02)	0.064	0.73 (0.45-1.18)	0.199
White	0.77 (0.53-1.12)	0.168	0.85 (0.55-1.29)	0.443
Marital status				
DSW	Reference		Reference	
Married	0.66 (0.51-0.87)	0.002	0.68 (0.51-0.9)	0.008
Single	0.83 (0.59-1.18)	0.301	0.69 (0.46-1.02)	0.064
Unknown	1.31 (0.79-2.17)	0.293	0.97 (0.52-1.8)	0.922
Median household income				
<50,000	Reference		Reference	
50,000-70,000	0.85 (0.67-1.08)	0.178	0.86 (0.66-1.12)	0.257
>70,000	0.99 (0.83-1.19)	0.915	1 (0.82-1.22)	0.975
Age				
<=62	Reference		Reference	
63-75	1.07 (0.85-1.35)	0.565	0.98 (0.76-1.27)	0.904
>=76	1.79 (1.36-2.34)	0	1.53 (1.12-2.07)	0.007
Grade				
I	Reference		Reference	
III: 72	1.02 (0.66-1.57)	0.931	0.98 (0.6-1.6)	0.925
III: 72	1.35 (0.88-2.08)	0.164	1.43 (0.88-2.31)	0.147
IV	1.03 (0.4-2.66)	0.945	1.07 (0.37-3.1)	0.894
Unknown	1.84 (1.25-2.7)	0.002	2 (1.3-3.08)	0.002
Summary stage				
Distant	Reference		Reference	
Localized	0.24 (0.13-0.43)	0	0.25 (0.13-0.46)	0
Regional	0.66 (0.41-1.06)	0.085	0.6 (0.36-1)	0.049
Unknown	0.6 (0.44-0.81)	0.001	0.53 (0.38-0.73)	0
AJCC Stage Group				
1	Reference		Reference	
2	1.34 (0.86-2.07)	0.196	1.7 (1.01-2.87)	0.044
3	1.25 (0.79-1.97)	0.333	1.82 (1.08-3.07)	0.024
4	3.27 (2.26-4.72)	0	4.29 (2.75-6.71)	0
Unknown	2.51 (1.65-3.82)	0	3.18 (1.93-5.24)	0
T				
T0	Reference		Reference	
T1	0.68 (0.27-1.7)	0.403	0.65 (0.23-1.82)	0.413
T2	0.53 (0.21-1.37)	0.189	0.52 (0.18-1.5)	0.228
T3	0.81 (0.33-1.99)	0.642	0.87 (0.32-2.39)	0.794
T4	0.75 (0.3-1.87)	0.533	0.85 (0.31-2.37)	0.763
TX	1.2 (0.49-2.94)	0.696	1.16 (0.42-3.17)	0.775
Unknown	0.99 (0.38-2.55)	0.978	0.84 (0.29-2.46)	0.752
N				
N0	Reference		Reference	
N1	1.09 (0.83-1.42)	0.539	1.24 (0.93-1.66)	0.151
N2	0.64 (0.35-1.19)	0.159	0.75 (0.39-1.43)	0.382
NX	1.78 (1.36-2.34)	0	1.9 (1.41-2.57)	0
Unknown	1.38 (0.92-2.06)	0.118	1.21 (0.75-1.96)	0.433
M				
M0	Reference		Reference	
M1	2.59 (2.05-3.28)	0	2.66 (2.05-3.45)	0
MX	2.14 (1.3-3.51)	0.003	2.38 (1.4-4.04)	0.001
Unknown	2 (1.33-3.01)	0.001	1.69 (1.04-2.75)	0.034

In multivariate regression analysis, older age (≥ 76 vs. ≤ 62, OR 1.37, p = 0.04) (where older age was found to be associated with poorer prognosis than younger age), tumor size (≤29mm vs ≥76mm/unknown OR 1.44 p=0.04) (where larger smaller tumor size was found to be associated with better prognosis than the larger tumor size)were determined as independent characteristics associated with OS of mucin group and year of diagnosis (2008–2013 OR 1.36, p=0.05) was determined as independent characteristic associated with CSS of mucin group. Meanwhile LNR (≤0.1 vs >0.3/unknown OR 4.3, p=0)(where lower LNR was found to be associated with better prognosis than higher LNR), chemotherapy (no chemotherapy/unknown vs. chemotherapy, OR 0.44, p = 0)(where receiving chemotherapy was associated with better prognosis than not receiving chemotherapy), number of primary tumors (one primary only vs. first of 2 or more primaries, OR 2.3, p = 0.007)(where less number of primary tumors was found to be associated with better prognosis than higher number of primary tumors), marital status (single vs married OR 0.5, p=0.002)(where being married was found to be associated with better prognosis than being single), summary stage (localized vs regional/unknown OR 0.17, p=0)(having localized tumor was associated with better prognosis than having regional metastasis) and AJCC stage group were determined as independent characteristics associated with both OS and CSS of mucinous cholangiocarcinoma. It was also noticed that LODDS, surgery, radiation therapy and tumor grade did not directly influence overall survival nor cancer specific survival (p ≥0.05).

### 3.4 Construction of predictive models for OS and CSS

**Figure 4:**
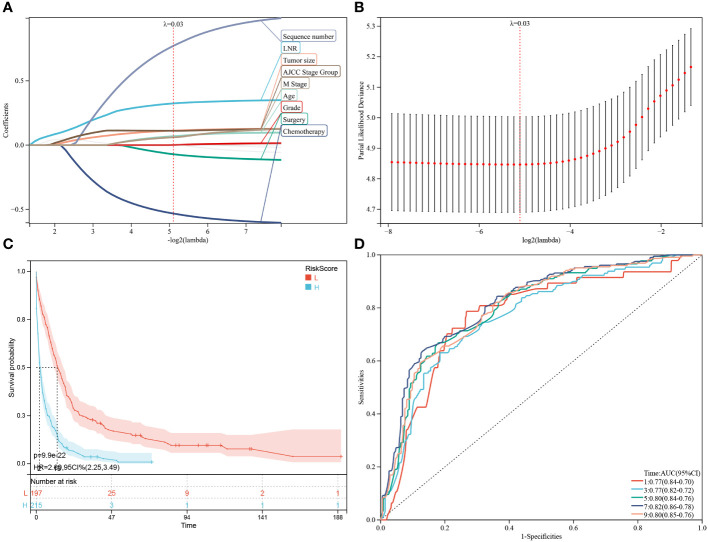
Construction and estimate of overall survival (OS)-associated predictive models. **(A, B)** The LASSO coefficient and deviance profiles represent the optimal λ value and risk factors, respectively. According to the risk score calculated by predictive models after PSM, KM curves of OS shown in **(C)** indicating that the prognosis of the low-risk group is significantly better than the high-risk score group. **(D)** According to the risk score, ROC curves of OS at 1, 3, 5, 7, 9 years was shown in the predictive model.

**Figure 5:**
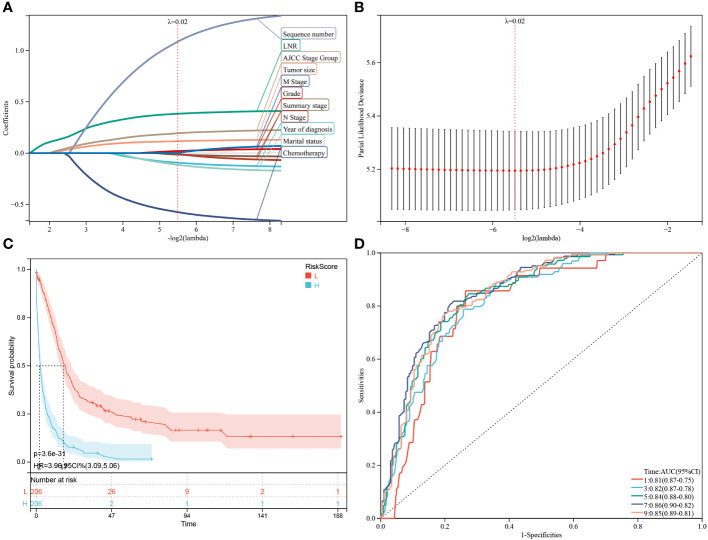
Construction and estimate of CSS-associated predictive models. **(A, B)** The LASSO coefficient and deviance profiles, represent the optimal λ value and risk factors, respectively. According to the risk score calculated by predictive models after PSM, KM curves of CSS shown in **(C)** indicating that the prognosis of the low-risk group is significantly better than the high-risk score group. According to the risk score in the predictive model, ROC curves of CSS at 1, 3, 5, 7, 9 years were shown in **(D)**.

Besides the aforementioned prognostic characteristics (**Table 3**), it is necessary to assess whether the adeno and mucinous cholangiocarcinoma groups can also predict the prognosis of patients. Therefore, LASSO-COX analysis was performed after univariate COX regression analysis to construct OS (**Figures 4A, B**) and CSS (**Figures 5A, B**) predictive models based on above-mentioned factors and groups. After 10-fold cross-validation, the optimal λ values 0.029 and 0.022 were obtained in OS and CSS models, respectively. It is worth noting that for non-hierarchical variables with more than 3 categories, these variables need to be converted into dummy variable matrix for analysis. Finally, the group and 9 prognostic factors were sifted for the predictive model of OS, including LNR, surgery status, chemotherapy status, tumor size, sequence number, age, tumor grade, AJCC stage group and metastasis M stage (**Figures 4A, B**).

**Table 3:** Multivariable Cox Regression for analyzing the associated factors for mucinous cholangiocarcinoma.

Subject characteristics	Overall survival (OS)		Cancer-specific survival (CSS)	
	HR (95%CI)	p value	HR (95%CI)	p value
Year of diagnosis				
<=2007			Reference	
2008-2013			1.36 (1-1.84)	0.051
>=2014			0.72 (0.51-1.03)	0.074
LNR				
<0.1	Reference		Reference	
0 1-0 3	1.26 (0.45-3.49)	0.659	1.43 (0.45-4.55)	0.544
>0 3	1.29 (0.39-4.27)	0.682	1.85 (0.47-7.22)	0.377
Unknown	2.89 (1.73-4.83)	0	4.3 (2.25-8.21)	0
LODDS				
<=-0 8	Reference		Reference	
>-0 8,<=-0 2	1.01 (0.43-2.39)	0.973	1.26 (0.45-3.57)	0.662
>-0 2	1.31 (0.37-4.68)	0.676	1 (0.23-4.3)	0.996
Unknown	NA	NA	NA	NA
Surgery				
No	Reference		Reference	
Yes	1.15 (0.62-2.12)	0.666	1.39 (0.71-2.73)	0.337
Radiation				
No	Reference		Reference	
Yes	0.8 (0.52-1.26)	0.338	0.75 (0.45-1.24)	0.26
Chemotherapy				
No/Unknown	Reference		Reference	
Yes	0.43 (0.33-0.56)	0	0.4 (0.3-0.53)	0
Tumor size				
<=29	Reference		Reference	
30-75	1.37 (0.91-2.06)	0.128	1.3 (0.82-2.07)	0.257
>=76	1.41 (0.83-2.38)	0.206	1.34 (0.74-2.43)	0.329
Unknown	1.44 (1-2.06)	0.047	1.42 (0.95-2.15)	0.09
Sequence number				
1st of 2 or more primaries	Reference		Reference	
One primary only	2.37 (1.27-4.43)	0.007	4.27 (1.9-9.63)	0
Marital status				
DSW	Reference		Reference	
Married	0.7 (0.52-0.94)	0.017	0.75 (0.54-1.04)	0.086
Single	0.65 (0.45-0.95)	0.027	0.5 (0.32-0.78)	0.002
Unknown	0.94 (0.55-1.62)	0.834	0.73 (0.37-1.42)	0.349
Age				
<=62	Reference		Reference	
63-75	0.97 (0.75-1.26)	0.838	0.86 (0.64-1.14)	0.283
>=76	1.37 (1.01-1.87)	0.046	1.16 (0.81-1.65)	0.425
Grade				
I	Reference		Reference	
II	0.89 (0.56-1.41)	0.617	0.79 (0.47-1.33)	0.365
III	0.97 (0.62-1.54)	0.905	0.89 (0.53-1.48)	0.654
IV	1.35 (0.48-3.77)	0.569	1.81 (0.55-5.94)	0.327
Unknown	0.99 (0.65-1.52)	0.97	1.1 (0.68-1.77)	0.697
Summary stage				
Distant	Reference		Reference	
Localized	0.19 (0.09-0.38)	0	0.17 (0.08-0.37)	0
Regional	0.91 (0.53-1.57)	0.746	0.71 (0.39-1.29)	0.26
Unknown	0.73 (0.52-1.03)	0.076	0.58 (0.4-0.85)	0.005
AJCC Stage Group				
1	Reference		Reference	
2	0.87 (0.52-1.47)	0.613	1.12 (0.61-2.07)	0.712
3	0.84 (0.48-1.48)	0.549	1.29 (0.66-2.5)	0.459
4	1.63 (0.99-2.7)	0.057	2.15 (1.18-3.9)	0.012
Unknown	0.76 (0.36-1.64)	0.49	1.32 (0.57-3.04)	0.517
N				
N0	Reference		Reference	
N1	1.1 (0.79-1.53)	0.574	1.17 (0.82-1.67)	0.389
N2	1.39 (0.61-3.17)	0.429	1.92 (0.76-4.81)	0.167
NX	1 (0.72-1.4)	0.977	0.96 (0.66-1.38)	0.825
Unknown	1.8 (0.87-3.74)	0.115	1.56 (0.68-3.56)	0.294
M				
M0	Reference		Reference	
M1	NA	NA	NA	NA
MX	2.22 (1.05-4.69)	0.036	2.21 (1-4.9)	0.05
Unknown	NA	NA	NA	NA

NA, not available.

Additionally, a survival study based on the risk score was performed on 412 individuals who had had PSM screening. For the OS model and the CSS model, the ideal cutoff value was calculated as 2.5165 and 2.7889, respectively. Based on their cutoff values, the included patients could be divided into high-risk and low-risk categories. The predictive model of OS was able to identify between patients with favorable or unfavorable prognoses, according to the Kaplan-Meier curve analysis. The high-risk group manifested a shorter OS than the low-risk group (p = 9.9e-22) (**Figure 4C**; **Supplementary Figure 1A**). Time-dependent ROC analysis showed that AUC of risk score for the prediction of 1,3,5,7,9-year OS was 0.77, 0.77, 0.80, 0.82 and 0.80, respectively (**Figure 4D**).

Subsequently, through the same modeling process, 11 prognostic factors were determined in the predictive model of CSS, including year of diagnosis, LNR, chemotherapy status, tumor size, sequence number, marital status, tumor grade, summary stage, AJCC stage group, lymph nodes status and metastasis M stage (**Figures 5A, B**). The high-risk group likewise manifested a shorter CSS than the low-risk group in **Figure 5C** (p = 3.6e-31) and **Supplementary Figure 1B** (p = 2.7e-23). Time-dependent ROC analysis showed that AUC of risk score for the prediction of 1,3,5,7,9-year CSS was 0.81, 0.82, 0.84, 0.86 and 0.85, respectively (**Figure 5D**).

### 3.5 Validation of predictive models for OS and CSS

**Figure 6:**
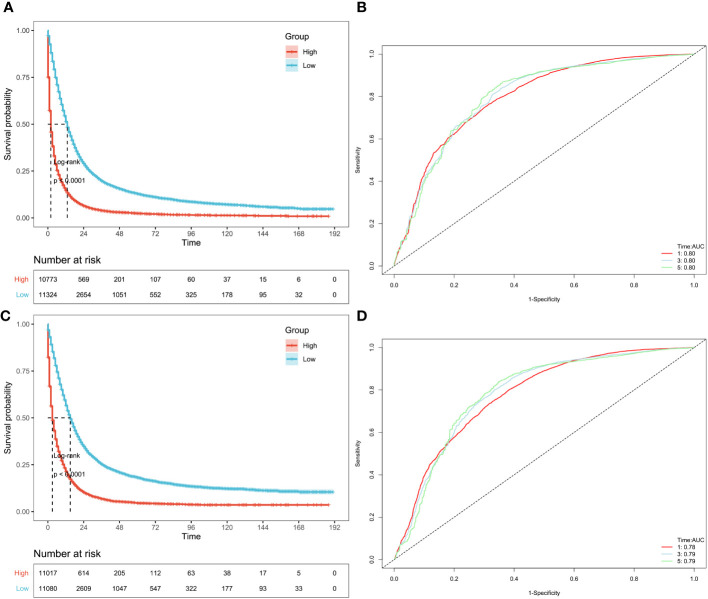
Validation of OS and CSS-associated predictive models in SEER datasets excluded by PSM. According to the risk score of the patients excluded by PSM, KM curves of OS **(A)** and CSS **(C)** indicating that the prognosis of the low-risk group is significantly better than the high-risk score group. And ROC curves of OS and CSS at 1,3,5 years were shown in **(B, D)**, respectively.

Firstly, validated the models by using samples excluded by PSM in the SEER database. The remaining 22097 patients excluded by PSM were again subjected to KM analysis. The included patients in predictive model of OS could be divided into high-risk (n=10773) and low-risk (n=11324) groups based on the median value of OS risk score. The Kaplan-Meier curve analysis revealed that the prognosis between high-risk group and low-risk group defined by predictive model of OS were significantly different. The high-risk group manifested a shorter OS i.e., 2 months than the low-risk group i.e., 13months (p < 0.0001) (**Figure 6A**; **Supplementary Figure 2A**). Furthermore, AUC based on time-dependent ROC of risk score for the prediction of 1,3- and 5-year OS was 0.80, 0.80 and 0.80, respectively (**Figure 6B**). Similarly, the included patients in predictive model of CSS could be classified into high-risk (n =11017) and low-risk (n =11080) groups through the median value of CSS risk score. Through the product-limited method (KM analysis), the high-risk group manifested a shorter CSS i.e., 3 months than the low-risk group i.e.,15momths (p < 0.0001), defined by the predictive model of CSS (**Figure 6C**; **Supplementary Figure 2B**). Time-dependent ROC analysis showed that AUC of risk score for the prediction of 1,3- and 5-year CSS was 0.78, 0.79 and 0.79, respectively (**Figure 6D**).

**Figure 7:**
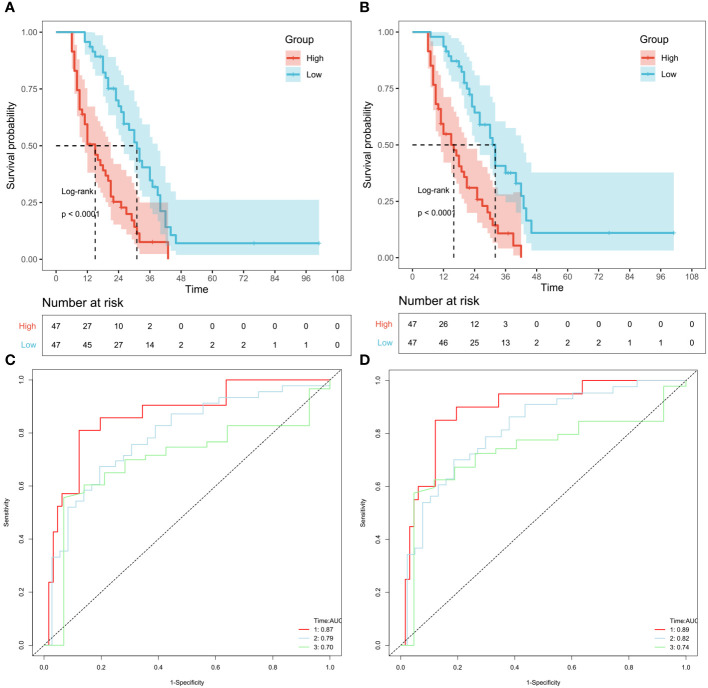
Validation of OS and CSS-associated predictive models in external datasets. According to the risk score of the patients excluded by PSM, KM curves of OS **(A)** and CSS **(B)** indicating that the prognosis of the low-risk group is significantly better than the high-risk score group. And ROC curves of OS and CSS at 1,2,3 years were shown in **(C, D)**, respectively.

Moreover, the external validation cohort comprised 94 patients with cholangiocarcinoma (adeno or mucinous), who were divided into high-risk and low-risk groups based on the optimal cutoff value of the risk score in the validation cohort. The Kaplan-Meier curve and the log-rank test were used to examine differences in survival between the two groups. In both the CSS prediction model and OS prediction model, patients in the low-risk group had a better prognosis than those in the high-risk group in the external validation cohort (OS and CSS: P<0.0001) (**Figures 7A, B**). Furthermore, the ROC curve showed that the AUC value of the OS prediction model at 1, 2, and 3 years were 0.87, 0.79, and 0.70, respectively, in the validation cohort (**Figure 7C**). Similarly, the AUC of the ROC in the CSS prediction model at 1, 2, and 3 years were 0.89, 0.82, and 0.74, respectively (**Figure 7D**).

### 3.6 Nomograms for OS and CSS using prognostic factors

**Figure 8:**
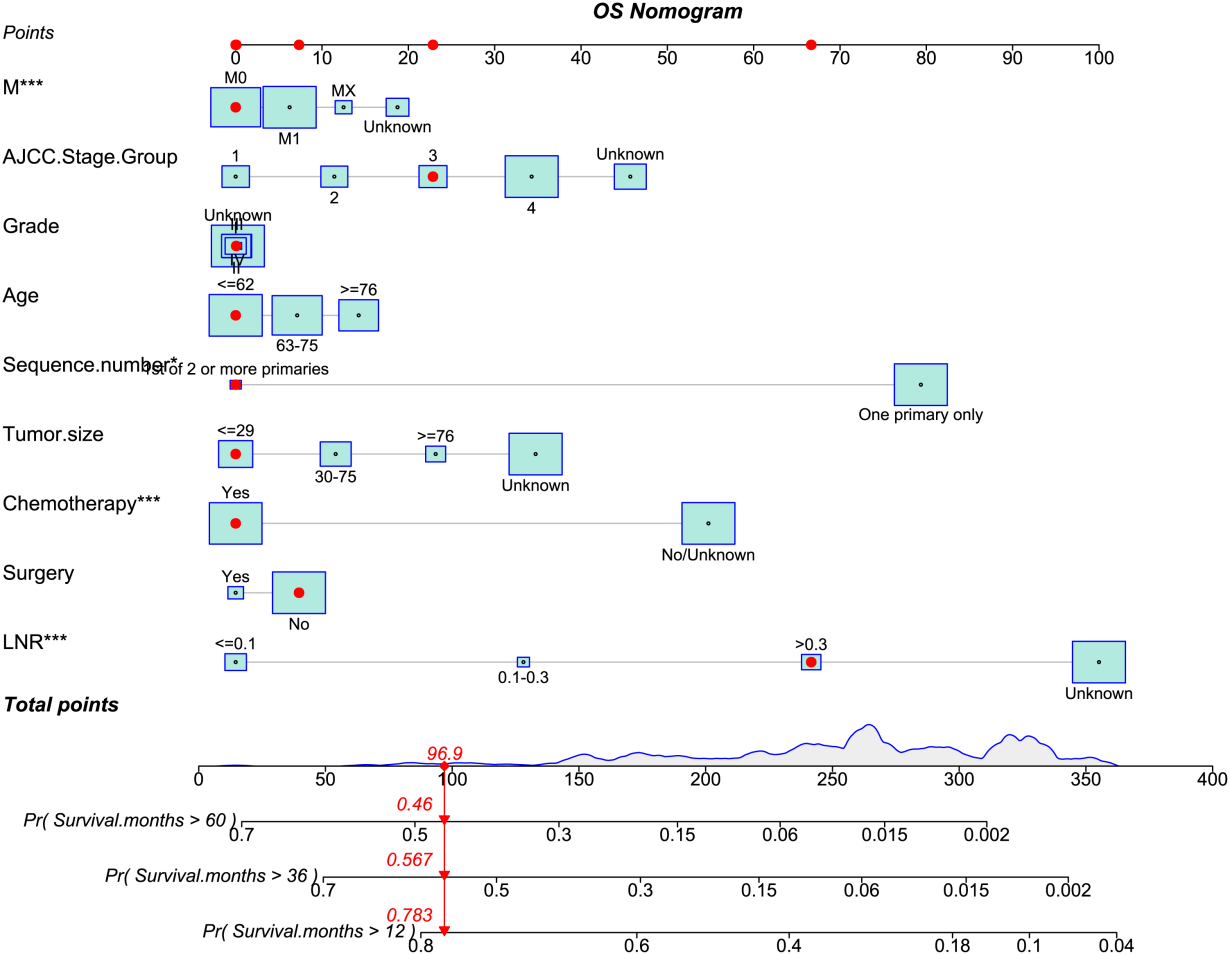
The nomogram of OS-associated predictive models. The sum of the scores indicated by the red arrows represents the survival probability corresponding to 1, 3, and 5 years.

In our investigation, nomograms were employed to enhance the practicality of the OS (**Figure 8**) or CSS (**Supplementary Figure 3**) predictive models by providing more vivid illustrations. Using the aforementioned scale, each characteristic’s score was calculated. The final score was determined as the total of these qualities’ scores. For patients with adeno and mucinous cholangiocarcinomas, we were able to forecast the prognosis of 1-, 3-, and 5-year OS or CSS using the perpendicular line connecting the total point axis and the two outcomes axis.

## 4 Discussion

Cholangiocarcinomas are a vast group of malignancies which are believed to have their origin in the epithelial cells either inside the liver or the biliary tract. These malignancies are usually hard to diagnose, their pathogenesis is not very well explained yet, their poor prognosis has been the main reason that their management has taken a nihilistic turn (1, 2).. A large proportion (90%) of biliary tract carcinoma are adenocarcinomas, with various histological subtypes, including adenocarcinoma (not otherwise specified), intestinal-type adenocarcinoma, papillary adenocarcinoma, and mucinous adenocarcinoma (3, 14, 15).

In the United States, the Surveillance, Epidemiology and End Results (SEER) database was formed and it contains population-based clinical survival data from registries that cover 34.6% of the country’s population. So, based on SEER database statistics, in this study we focused to develop a prognostic nomogram which can be used widely for adenocarcinomas as well as rare variant i.e., mucinous cholangiocarcinoma.

Previously reported studies have mostly been focusing on location of tumor as N. A. van der Gaag et al., in 2012 introduced a prognostic nomogram for patients which were undergoing resection of extrahepatic cholangiocarcinoma. According to them, tumor location of extrahepatic CCA does not independently predict CSS after resection and constructed a prognostic model based on lymph node status, residual tumor levels of resection margins, and tumor differentiation status, that predicted CSS better than TNM staging (16). But this study is limited only for extrahepatic tumors and did not give any clues if these outcomes apply to different pathological subvariants. Also, Alfredo Guglielmi et al., in 2009 published a study of 81 patients from Italy and claimed that only radical resection of ICC can achieve long-term survival. They stated that best outcomes are seen in patients who underwent R0 resection for tumors without lymph node metastasis or vascular invasion. Lymph node metastases, vascular invasion, and histologic type are significant prognostic indicators associated with poor survival. In order to achieve better outcomes for these individuals, different therapeutic modalities (such as adjuvant or neoadjuvant therapy) should be considered (17). They claimed lymph node status to be the most important prognostic factor after resection. These findings are consistent in our study too such as we also found that lymph node status as our analysis also states that having less number of LNR is associated with better prognosis than larger LNR, tumor grade where low tumor grade is associated with better prognosis than higher tumor grade and tumor size where smaller tumor size is associated with better prognosis than larger tumor size and these are independently significant in prediction of prognosis. We also found six more factors associated with prognosis in aim to provide a broader and clearer clinical picture to the clinicians which will help to improve the clinical management and prediction of prognosis in these patients.

The cancer prognostic factors are of great importance in relation to anticipate the future risk, evaluation of recovery chances and recurring of the disease (18). In the present study, we employed the process of data mining of CCA patients using SEER cancer database of the National Cancer Institute (http://www.seer.cancer.gov), released in November 2021 via SEER*Stat software (version 8.4.0.1). Meanwhile, SEER cancer database allows data availability from 17 population-based cancer registries in the United States. For case selection, several factors such as (1) the tumor was not primary and (2) the case was lacking complete follow-up data, were assimilated to determine the exclusion criteria. The impartial clinical information including patient ID and age, tumor characteristics, quantitative data of primary tumors, lymph nodes status, surgery and chemotherapy of every patient was also retrieved from SEER database. Based on the comprehensive analysis of CCA patients’ data, this study clearly concludes that there is not any significant difference in prognosis of mucinous CCA and ordinary CCA. We found that older age was found to be associated with poorer prognosis than younger age, larger smaller tumor size was found to be associated with better prognosis than the larger tumor size and were determined as independent characteristics associated with OS of mucin group and year of diagnosis was determined as independent characteristic associated with CSS of mucin group. Meanwhile lower LNR was found to be associated with better prognosis than higher LNR, receiving chemotherapy was associated with better prognosis than not receiving chemotherapy, less number of primary tumors was found to be associated with better prognosis than higher number of primary tumors, being married was found to be associated with better prognosis than being single, having localized tumor was associated with better prognosis than having regional metastasis and AJCC stage group were determined as independent characteristics associated with both OS and CSS of mucinous cholangiocarcinoma. It was also noticed that LODDS, surgery, radiation therapy and tumor grade did not directly influence overall survival nor cancer specific survival. However, all other clinical information (described previously) are independent key factors in prognosis and OS of the mucinous and ordinary CCA patients. In addition, we developed a novel prognostic model and nomogram to be used in the management of both types of CCA (mucinous and adeno CCA) in a parallel time frame.

Most patients with CCA are diagnosed at advanced phase of the disease and have poor OS (19–22), however, its therapeutic and diagnostic targets are still unknown. In an effort to find a more useful prospective diagnostic target for CCA, we constructed and validated a prognostic model and nomogram. Initially we screened 22509 CCA patients including 22299 with adeno CCA and 210 with mucinous CCA using SEER database and collected their clinical information including age, year of diagnosis, LNR, LODDS, tumor size, sequence number, summary stage, and AJCC stage group. The baseline clinical characteristics such as total number of tumors, race, median household income, and marital status between mucinous and adeno CCA patients were found to be similar (P>0.05). Then, the PSM analysis was incorporated to omit the bias selection between with mucinous and adeno CCA patients. Meanwhile the Kaplan-Meier survival analysis allowed us to determine the percentage of patients who survive a specific event (23). Before and after the PSM analysis, the patients were assessed for Kaplan-Meier survival analysis and showed no significant difference. The Kaplan-Meier survival analysis prior to the PSM analysis was performed on a total of 22509 patients including 22299 with adeno CCA and 210 with mucinous CCA. We found that both groups (mucinous and adeno CCA) have similar median OS i.e., 2.0 months. However, the median CSS was different in both groups as adeno CCA group (2 months) and mucinous CCA group (3.0 months). Overall, there was a non-significant difference for both OS and CSS rate between adeno CCA and mucinous CCA groups (**Figures 3A-D**).

To avoid overfitting of the prediction model and to solve clinical decision problems, the established and constructed nomogram’s predictive accuracy and predictive validity were thoroughly assessed while finding prognostic markers (24). The relationship between CCA patients’ prognostic factors and their relative effects on OS and CSS were determined using univariate and multivariate Cox regression analyses. Meanwhile, univariate and multivariate analysis helps to explore the distribution frequency of an independent key factor between both groups of CCA patients (18). In this study, the univariate regression analysis showed similar prognostic factors for both OS and CSS in mucinous CCA group. However, multivariate regression analysis revealed independent set of prognostic variables as risk factors associated with OS and CSS of mucinous CCA group. In addition, we observed that LODDS, surgery, radiation therapy and tumor grade did not directly influence OS nor cancer specific survival (p ≥ 0.05). In addition, the predictive OS and CSS models were constructed using LASSO-COX analysis. Anyhow, LASSO-COX analysis is a durable tool for data analysis and integrating the significant prognostic factors as well (25). Following the 10-fold cross-validation, optimum λ values as 0.029 and 0.022 were obtained in OS and CSS models, respectively. Finally, the group and 9 prognostic factors including LNR, surgery status, chemotherapy status, tumor size, sequence number, age, tumor grade, AJCC stage group and metastasis M stage were determined in the predictive model of OS. Besides, depending upon the risk score, PSM analysis patients (n = 412) were exposed to survival analysis and at standard cut off values given as OS model = 2.5165 and CSS model = 2.7889. This analysis allowed us to segregate the patients into two groups as high-risk and low-risk groups. Meanwhile, The Kaplan-Meier curve analysis revealed that predictive OS model distinguished patients with good or bad prognoses. The predictive model of CSS showed 11 prognostic factors affiliated with the pathogenesis of CCA. Next, to validate the OS and CSS predictive models, we incorporated PSM excluded patients (n=22097) data to survival analysis based upon their risk score. We developed two groups of patients as high-risk (n = 10773) and low-risk (n = 11324) groups based on their median value in OS predictive model. The high-risk group manifested a shorter OS i.e., 2 months than the low-risk group i.e., 13months with significant statistical difference (p < 0.0001) (**Figure 6A**; **Supplementary Figure 2A**). Time-dependent ROC analysis showed that AUC of risk score for the prediction of 1,3 and 5-year OS was 0.80, 0.80 and 0.80, respectively (**Figure 5B**). Also, the OS predictive model discriminated the patients with good or bad prognoses. Correspondingly, CSS predictive model also helped us to classify the patients into high-risk (n = 11017) and low-risk (n = 11080) groups based on their median value. The high-risk group manifested a shorter CSS i.e., 3 months than the low-risk group i.e.,15 months (p < 0.0001) (**Figure 6C**; **Supplementary Figure 2B**). Time-dependent ROC analysis showed that AUC of risk score for the prediction of 1,3- and 5-year CSS was 0.78, 0.79 and 0.79, respectively (**Figure 5D**). Here, we examined that the patients in high-risk groups had poor prognosis and shorter OS and CSS than low-risk groups.

While encountering the risk factors permitted us to explore and anticipate the future risk of the disease. To best of our knowledge, we attempt to deliver a significant and more practical prognostic model and nomogram for mucinous and adeno CCA. In conclusion, it is stated that there is no difference in prognosis between mucinous CCA and ordinary CCA. Cumulatively, all prognostic variables such as age tumor size, the number of primary tumors, AJCC stage, Grade, lymph node status, metastasis, surgery and chemotherapy are the independent factors in prognosis and overall survival of the patients with mucinous and ordinary CCA. So, our study will give future insights about the clinical management of adeno and mucinous cholangiocarcinoma and help the clinicians to rely on these prognostic factors while managing these patients and assessing prognosis based on these factors. As recent studies are focusing on finding out prognostic biomarkers for cholangiocarcinoma, it would be a favorable idea to update our model by also including prognostic biomarkers cholangiocarcinoma to further enhance its accuracy and applicability.

## Data availability statement

The original contributions presented in the study are included in the article/**Supplementary Material**. Further inquiries can be directed to the corresponding authors.

## Ethics statement

The studies involving humans were approved by ethics committee of Xiangya Second Hospital, Central South University. The studies were conducted in accordance with the local legislation and institutional requirements. Written informed consent for participation was not required from the participants or the participants’ legal guardians/next of kin in accordance with the national legislation and institutional requirements. The manuscript presents research on animals that do not require ethical approval for their study.

## Author contributions

MA: Writing – original draft. Z-jZ: Writing – original draft. Z-tL: Writing – original draft. Y-pH: Writing – original draft. Y-xW: Writing – original draft. HZh: Writing – original draft. LX: Writing – review & editing. YW: Writing – review & editing. HZo: Writing – review & editing.
