# Supplementary figures and images for: Prognostic models for mucinous and non-specific adeno cholangiocarcinoma: a population-based retrospective study
Source: Front Endocrinol (Lausanne). 2024 Jun 11;15:1284283. doi: 10.3389/fendo.2024.1284283 (PMC11196395; doi:10.3389/fendo.2024.1284283)

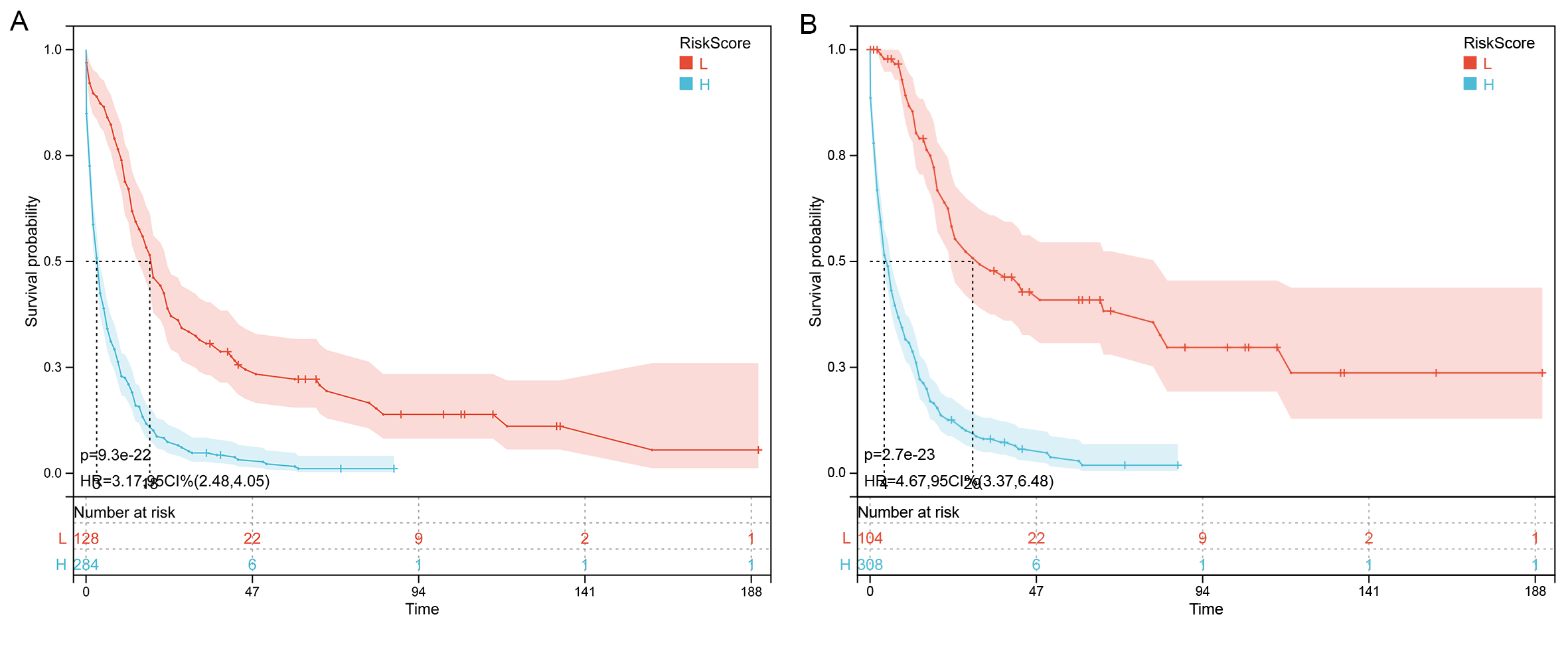

Supplement: Supplementary file 1 [file Image_1.tif]

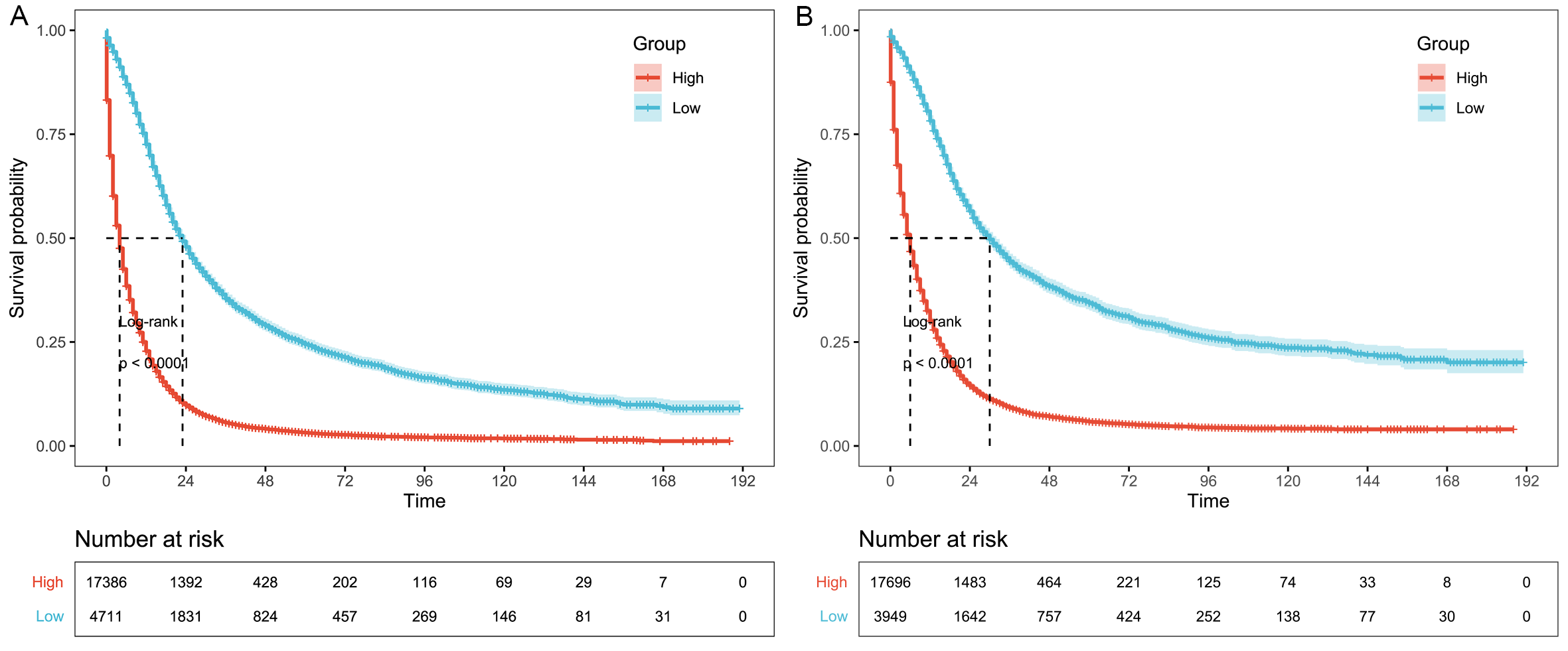

Supplement: Supplementary file 2 [file Image_2.tif]

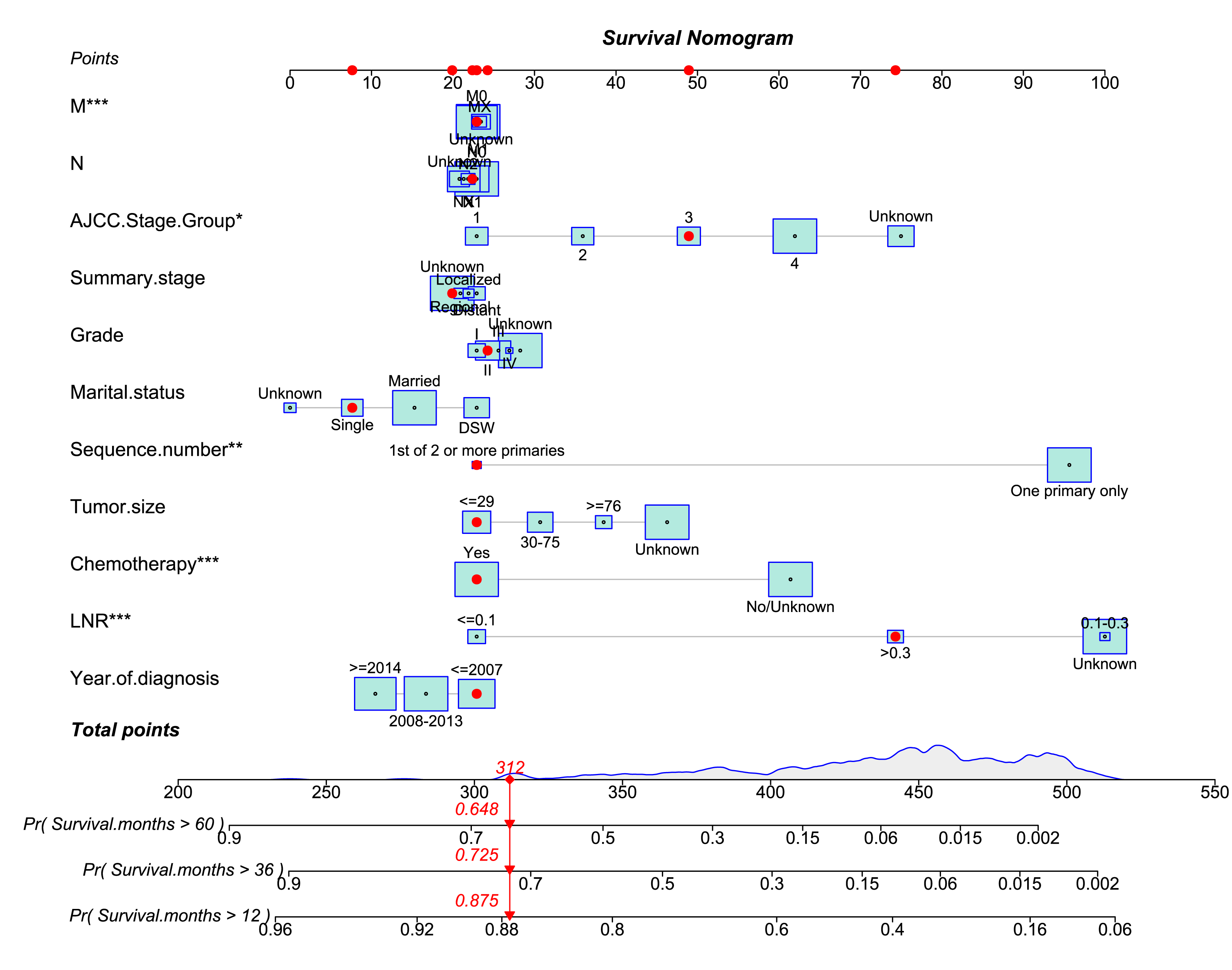

Supplement: Supplementary file 3 [file Image_3.tif]
